# A Multi-Biochemical and In Silico Study on Anti-Enzymatic Actions of Pyroglutamic Acid against PDE-5, ACE, and Urease Using Various Analytical Techniques: Unexplored Pharmacological Properties and Cytotoxicity Evaluation

**DOI:** 10.3390/biom9090392

**Published:** 2019-08-21

**Authors:** Miroslava Šudomová, Sherif T. S. Hassan, Haroon Khan, Mahsa Rasekhian, Seyed Mohammad Nabavi

**Affiliations:** 1 Museum of literature in Moravia, Klášter 1, 664 61 Rajhrad, Czech Republic; 2 Department of Natural Drugs, Faculty of Pharmacy, University of Veterinary and Pharmaceutical Sciences Brno, Palackého tř. 1946/1, 612 42 Brno, Czech Republic; 3 Department of Pharmacy, Abdul Wali Khan University, Mardan 23200, Pakistan; 4 Pharmaceutical Sciences Research Center, Health Institute, Kermanshah University of Medical Sciences, Kermanshah 6734667149, Iran; 5 Applied Biotechnology Research Center, Baqiyatallah University of Medical Sciences, Tehran 14359-16471, Iran

**Keywords:** pyroglutamic acid, anti-enzymatic properties, phosphodiesterase 5, angiotensin-converting enzyme, urease, ESI-mass spectrometry, cytotoxicity

## Abstract

In the current study, pyroglutamic acid (pGlu), a natural amino acid derivative, has efficiently inhibited the catalytic activities of three important enzymes, namely: Human recombinant phosphodiesterase-5A1 (PDE5A1), human angiotensin-converting enzyme (ACE), and urease. These enzymes were reported to be associated with several important clinical conditions in humans. Radioactivity-based assay, spectrophotometric-based assay, and an Electrospray Ionization-Mass Spectrometry-based method were employed to ascertain the inhibitory actions of pGlu against PDE5A1, ACE, and urease, respectively. The results unveiled that pGlu potently suppressed the activity of PDE5A1 (half-maximal inhibitory concentration; IC_50_ = 5.23 µM) compared with that of standard drug sildenafil citrate (IC_50_ = 7.14 µM). Moreover, pGlu at a concentration of 20 µg/mL was found to efficiently inhibit human ACE with 98.2% inhibition compared with that of standard captopril (99.6%; 20 µg/mL). The urease-catalyzed reaction was also remarkably inactivated by pGlu and standard acetohydroxamic acid with IC_50_ values of 1.8 and 3.9 µM, respectively. Remarkably, the outcome of in vitro cytotoxicity assay did not reveal any significant cytotoxic properties of pGlu against human cervical carcinoma cells and normal human fetal lung fibroblast cells. In addition to in vitro assays, molecular docking analyses were performed to corroborate the outcomes of in vitro results with predicted structure–activity relationships. In conclusion, pGlu could be presented as a natural and multifunctional agent with promising applications in the treatment of some ailments connected with the above-mentioned anti-enzymatic properties.

## 1. Introduction

Recently, investigations on enzyme inhibition have gained great attention in both research and industrial fields, where such researches have led to imperative findings of important biomolecules used in the treatment of many diseases [1]. Natural products represent a rich source of secondary metabolites with a broad range of therapeutic activities, where such metabolites have shown to exert decreased undesirable effects, less resistance, and various mechanisms of action compared to synthetic drugs [2]. Therefore, their use has become popular in natural-based medicine in many countries all over the world [3].

Pyroglutamic acid (pGlu; 5-oxoproline or pidolic acid) is a naturally occurring and little investigated amino acid derivative that can be formed enzymatically or non-enzymatically. This compound was observed to be widely synthesized in diverse living cells reported from archaebacteria to humans [4]. Moreover, pGlu was detected either as a free acid or bound at the N terminus of proteins and peptides and contributes as a biological intermediate agent in numerous chemical pathways. Up to now, there are no clinically accepted and/or marketed medicine that contains pGlu as an active constituent for any prescribed therapeutic indication. On the other hand, pGlu is commercially available as a non-prescription nootropic dietary supplement [5,6]. Previous studies stated that pGlu induced in vivo anti-diabetic properties and inhibited in vitro energy production and lipid synthesis in the cerebral cortex of young rats [7,8].

Phosphodiesterase type 5 (PDE-5) is a cyclic guanosine monophosphate (cGMP)-specific enzyme that is largely scattered in smooth muscle. This enzyme was detected in bovine lung, rat platelets corpus cavernosum, brain, lung, liver, heart, platelets, stomach, prostate, urethra, and bladder [9,10,11,12]. Since PDE-5 plays an essential role in vascular relaxation arbitrated by the NO/cGMP pathway in vascular smooth muscle cells, this enzyme has been deemed to be a leading target for the development of inhibitors used in the therapy of diseases linked with decreased cGMP levels [13,14].

Angiotensin-converting enzyme (ACE) is a zinc-dependent dicarboxypeptidase with two catalytic domains. ACE has shown to play a critical role in blood pressure regulation by converting angiotensin I to angiotensin II and was detected in numerous cells including neuroepithelial, endothelial, epithelial, and immune system cells [15,16]. In addition to angiotensin I, ACE cleaves several peptides and hence alters various physiological functions, including renal development and male reproduction [15]. Based on the above-mentioned multifunctional capabilities, ACE has been considered to be a striking target for drug design [17]. 

Urease (urea amidohydrolase), an enzyme that catalyzes the hydrolysis of urea to produce ammonia and CO_2_, impulsively hydrolyzes to yield H_2_CO_3_ and a second molecule of ammonia at a rate nearly 10^14^ times the rate of the uncatalyzed reaction [18]. As a result of such a reaction, imperative damaging effects on humans and agriculture are taking place due to the high concentrations of ammonia produced from this reaction along with the increasing rate of pH [19].

Urease was found in several organisms such as plants, bacteria, fungi, and algae, where this enzyme was reported to be a substantial virulence factor connected with the pathogenesis of several clinical conditions, such as pyelonephritis, urolithiasis, ammonia, and hepatic encephalopathy, hepatic coma and urinary catheter encrustation, and formation of infection-induced urinary stones [20,21]. Thus, to treat such diseases, urease has been considered to be a significant target for urease inhibitor (UI) drug design [22].

## 2. Materials and Methods

### 2.1. Anti-PDE5A1 Inhibitory Properties

#### 2.1.1. Protein Expression and Purification

The expression and purification of PDE5A1 were assayed following the previously published protocol [23]. Briefly, the human catalytic domain coding of PDE5A1 (residues 535−860; obtained from Motol University Hospital (MUH), Prague, Czech Republic) was cloned to vector pET15b (MUH, Prague, Czech Republic), then the recombinant plasmid (pET15b-PDE5A1) was transported to *E. coli* strain BL21 (MUH, Prague, Czech Republic) for overexpression. Further, the *E. coli* cell conveying the plasmid (pET15- PDE5A1) was cultivated in Lysogeny Broth medium at 37 °C to reach absorbance (optical density; OD_600_ = 0.7), followed by adding isopropyl *β*-_D_-thiogalactopyranoside (IPTG; 0.1 mM) to inspire expression for the additional 40 h at 15 °C. 

The purification of PDE5A1 protein (9.84 mg yielded from 2-L of cell culture with a purity >95%) was achieved using a sodium dodecyl sulfate-polyacrylamide gel electrophoresis (SDS−PAGE) supplied with Ni-NTA column (⌀ = 2.5 cm, 15 mL QIAGEN agarose beads, GE Healthcare, Buckinghamshire, UK), Q-column (⌀ 2.5 cm × 8 cm, GE Healthcare, Buckinghamshire, UK), and Superdex 200 column (⌀ 2.5 cm × 45 cm, GE Healthcare, Buckinghamshire, UK).

#### 2.1.2. Anti-PDE5A1 Activity

PDE5A1 activity was assayed, as described earlier, with minor modification [24]. Briefly, PDE5A1 (75.3 µg/mL) prepared in Tris–HCl (50 mM; pH = 8.0), MgCl_2_ (10 mM) and DTT (1 mM) was mixed with 10–30 nM of ^3^H-cGMP (20,000–30,000 cpm/assay, GE Healthcare, Buckinghamshire, UK) as a substrate as well as pGlu (Sigma-Aldrich, Berlin, Germany; purity ≥99%) and sildenafil citrate (Sigma Aldrich, Berlin, Germany; European Pharmacopoeia (EP) reference standard) as inhibitors (at concentrations ranging from 3 to 10 µM). The enzymatic reaction took place at 25 °C for 20 min and then the reaction was stopped by adding ZnSO_4_ (0.2 N). Further, the reactant ^3^H-GMP was accelerated by Ba(OH)_2_ (0.2 N), while unreacted ^3^H-cGMP was kept in the supernatant. PerkinElmer 2910 liquid scintillation counter (Thermo Fisher Scientific, Paisley, UK) was utilized to measure the radioactivity of the supernatant through 2.5 ml Ultima Gold liquid scintillation cocktails (Thermo Fisher Scientific, Paisley, UK). The half-maximal inhibitory concentration (IC_50_) values for test inhibitors were ascertained in the presence of proper concentrations of ^3^H-cGMP (10–30 nM) and the enzyme (75.3 µg/mL) that hydrolyze up to 70% of the substrate and subsequently calculated by nonlinear regression analysis. Each measurement was conducted in triplicate. 

### 2.2. Anti-Angiotensin-Converting Enzyme (ACE) Properties

Spectrophotometric-based assay was used to ascertain the enzymatic activity of human ACE following the previously described method [25]. Briefly, the ACE-catalyzed reaction is based on the formation of hippuric acid as a product, where 166 mU/mL of human ACE (Sigma-Aldrich, Prague, Czech Republic) mixed with Tris Buffer (50 mM, pH = 8.3) was incubated with pGlu (Sigma-Aldrich, Berlin, Germany; purity ≥99%) and captopril (Sigma Aldrich, Prague, Czech Republic; EP reference standard) at a concentration of 20 µg/mL for 80 min at 37 °C. Subsequently, the prepared solution was blended with 110 μL of the substrate hippuryl-histidyl-leucine (10 mM; Sigma-Aldrich, Prague, Czech Republic). The reaction product hippuric acid was inspected at λ 228 nm using UV–VIS spectrophotometer (SPECTROstar^Nano^ BMG Labtech, Ortenberg, Germany) and the % inhibition was determined.

### 2.3. Anti-Urease Properties

#### 2.3.1. Enzyme, Substrate, Inhibitors, and Chemicals

Jack bean urease (JBU) from *Canavalia ensiformis*, urea, and acetohydroxamic acid (AHA; standard urease inhibitor) were acquired from Sigma-Aldrich, Prague, Czech Republic), whereas pGlu was obtained from Sigma–Aldrich, Berlin, Germany with purity ≥99%. All used chemical reagents were gained from commercial suppliers without additional refinement.

#### 2.3.2. Instrumentation: ESI-MS

A system pump-injector (Agilent 1200, Berlin, Germany) attached with a Sciex-3200QTRAP–hybrid triple quadrupole/linear ion trap mass spectrometer (MS; Toronto, ON, Canada) joined with Electrospray Ionization (ESI) was used. In order to perform the analysis, ESI-MS runs without an HPLC column using a flow injection analysis (FIA) mode along with optimized operational parameter settings such as curtain gas (CUR), 25 psi; nebulizer gas (GS1), 50; auxiliary gas (GS2), 40; declustering potential (DP), 15 V; ion spray voltage, −4000 V; turbo temperature, 450 °C. For detection and quantitation of urea (*m*/*z* 61→44), MS in positive ion mode was operated in multiple reaction monitoring (MRM) analysis. Mobile phases such as HCOOH (0.1%) and HCOONH_4_ (1 mM) were used with the flow rate established at 0.5 mL/min along with the injection volume (10 μL) [26].

#### 2.3.3. Determination of Anti-Urease Activity

The catalytic activity of urease was assessed using an Electrospray Ionization-Mass Spectrometry (ESI-MS) based method, as previously developed, validated based on repeatability and stability studies, and described by Hassan et al. [26]. It is known that enzyme activity could be detected through the depletion of substrate or formation of product. Accordingly, the principal mechanism of the method is focusing on the monitoring of the urease-catalyzed reaction through the reduction of urea (substrate) concentration in the presence and absence of inhibitors. Briefly, a solution contains JBU (34.7 µg/mL) prepared in HCOONH_4_ buffer (1 mM; pH = 7.6) was incubated with pGlu (15 μM) and AHA (15.2 μM) for 20 Minutes to attain binding equilibrium. Further, urea (272 µM) was added to the solution mixture. The obtained solution was further injected into the FIA system and the concentration changes of urea were observed. Subsequently, the analysis of the kinetics of urea depletion by ESI-MS was achieved by integrating areas (total counts) under peaks for urea in the FIA system. IC_50_ values for test inhibitors were determined following the above-mentioned method [26]. In order to evaluate the repeatability of measurements, we conducted multiple measurements of enzymatic reaction of the same sample. The precision of time-course analysis was calculated as the relative standard deviation (RSD; %) of multiple measured slopes.

### 2.4. Cytotoxicity Study

#### 2.4.1. Cell Lines, Medium and Reagents

Human cervical carcinoma cells (HeLa-R2) and normal human fetal lung fibroblast cells (MRC-5) were acquired from MUH, Prague, Czech Republic. Concisely, the cells as monolayer culture were cultivated in a culture medium (Roswell Park Memorial Institute RPMI;1640; Sigma Chemicals Co., Saint Louis, MO, USA) supplemented with 4-(2-hydroxyethyl) piperazine-1-ethanesulfonic acid (HEPES) (25 mM), 10% of heat-inactivated fetal calf serum (FCS; pH = 7.2), penicillin (192 U/mL), streptomycin (200 mg/mL), and _L_-glutamine (3 mM). Further, the test cells were grown in the humidified state with 5% CO_2_ at 37 °C, and then sub-cultured twice for 7 days, as previously designated [27].

#### 2.4.2. Assessment of Cytotoxicity

MTT assay (3-(4,5-dimethylthiazol-2-yl)-2,5-diphenyltetrazolium Bromide) (Sigma-Aldrich, Berlin, Germany) was applied to determine the potential cytotoxic effect of pGlu (stock solutions of pGlu were prepared in dimethyl sulfoxide (DMSO; 1%) and then diluted with nutrient medium to the final concentrations up to 200 µg/mL) on human cervical carcinoma cells (HeLa-R2) and normal human fetal lung fibroblast cells (MRC-5), as previously detailed [27]. Cisplatin (stock solutions were prepared in 0.9% NaCl and then diluted with nutrient medium to the final concentrations up to 10 µg/mL), a standard anticancer medication was chosen as a reference control (Sigma-Aldrich, Prague, Czech Republic; EP reference standard). A microplate reader (Infinite M200, Tecan, Salzburg, Austria) was adapted to detect the absorbance of test samples at λ 570 nm. The cell survival diagrams that demand to impede 50% of cell survival were acquired to assess the IC_50_ values of test compounds.

### 2.5. Molecular Docking Studies 

#### Protein-Ligand Preparation and Performance of Docking Studies

The RCSB Protein Data Bank (www.rcsb.org) was employed to retrieve the 3D-crystal structure of phosphodiesterase 5A1 (PDE5A1) catalytic domain in complex with sildenafil (PDB ID: 2H42), 3D-crystal structure of human angiotensin-converting enzyme (ACE) docked with captopril (PDB ID: 1UZF), 3D-crystal structure of jack bean urease (JBU; PDB ID: 3LA4), and 3D-structure of pGlu (SDF file ID: PCA). PyRx docking software fitted with Autodock VINA (version 0.8, The Scripps Research Institute, La Jolla, CA, USA) was exploited to accomplish the molecular docking studies and to assess the binding modes of pGlu in the active sites of the above-mentioned enzymes.

To ascertain the optimal parameters for reliable docking analyses, sildenafil was extracted from the 3D-crystal structure of (PDB ID: 2H42) and further re-docked back into the crystal structure of the enzyme, while captopril was erased from the 3D-crystal structure of (PDB ID: 1UZF) and re-docked back into the enzyme. All optimal parameters, settings, calculations, protonation conditions, and the overall charges were tracked, as previously designated [28,29]. Additionally, Zn^+2^ and Mg^+2^ ions were assigned during the processing of docking analysis for PDE5A1. All graphical presentations of the docked complexes were illustrated using Discovery studio visualizer version v19.1.0.18287 (BIOVIA, San Diego, CA, USA) [30].

## 3. Results and Discussion

### 3.1. Assessment of Anti-PDE5A1 Activity

The activity of pGlu against human recombinant PDE5A1 is presented in Table 1, where pGlu strongly inhibited the enzyme in a significant manner with an IC_50_ value of 5.23 µM compared with that of sildenafil citrate (IC_50_ = 7.14 µM). It is noteworthy that additional studies are required to be performed against all PDE families to verify the selectivity of pGlu against PDE5A1. Moreover, the mechanism by which pGlu induced anti-PDE5A1 activity should be explored in further investigations. 

Presently, there are several clinically effective PDE-5 inhibitors used to treat erectile dysfunction, such as sildenafil, vardenafil, tadalafil, avanafil, udenafil, and mirodenafil [31,32,33], while sildenafil and tadalafil are being used to cure pulmonary arterial hypertension [34]. Numerous studies have conveyed severe side effects of these inhibitors, especially with sildenafil, where the problems with vision disturbance, hearing loss, and headache were claimed [35,36]. Therefore, there is an urgent need to explore novel, selective, and effective PDE-5 inhibitors with reduced adverse effects to overcome such complications [37]. To the best of our knowledge, this is a first study on the inhibition efficacy of pGlu against PDE-5. 

### 3.2. Assessment of Anti-ACE Activity

Over the past few decades, the inhibition of ACE has played a significant role in regulating hypertension and linked diseases. As shown in Table 2, pGlu had a concentration-dependent activity on human ACE, where pGlu possessed a remarked inhibitory property against the enzyme via decreasing the amount of hippuric acid formed with 98.2% of inhibition (at concentration of 20 µg/mL) compared to captopril (99.6% at a concentration of 20 µg/mL). 

Hypertension is a long-term medical condition and has been considered as a risk factor for many illnesses such as cardiovascular disease, heart failure, and kidney disease. Given the vital role of ACE in regulating blood pressure, controlling the activity of this enzyme is very significant in curing cardiovascular system-related diseases [38]. Currently, several antihypertensive drugs are available on the market such as captopril, lisinopril, alacepril, and benazepril with reported undesirable effects [17,39]. On the other hand, synthetic ACE inhibitors were found to elicit unwanted effects on humans [40]. Thus, it is crucial to search for alternative sources of ACE inhibitors, such as natural products, that offer reduced undesirable effects and various mechanisms of action. Natural-derived molecules such as polyphenols, flavonoids, tannins, and peptides were testified in several investigations with inhibitory effects on ACE [41,42,43,44]. Thus far, no studies have declared the capability of pGlu to reduce the risk of hypertension and its associated ailments by suppressing the enzymatic action of ACE. Therefore, in this study, we assert a first report on the inactivation action of pGlu against ACE.

### 3.3. Evaluation of Anti-Urease Activity

**Figure 1:**
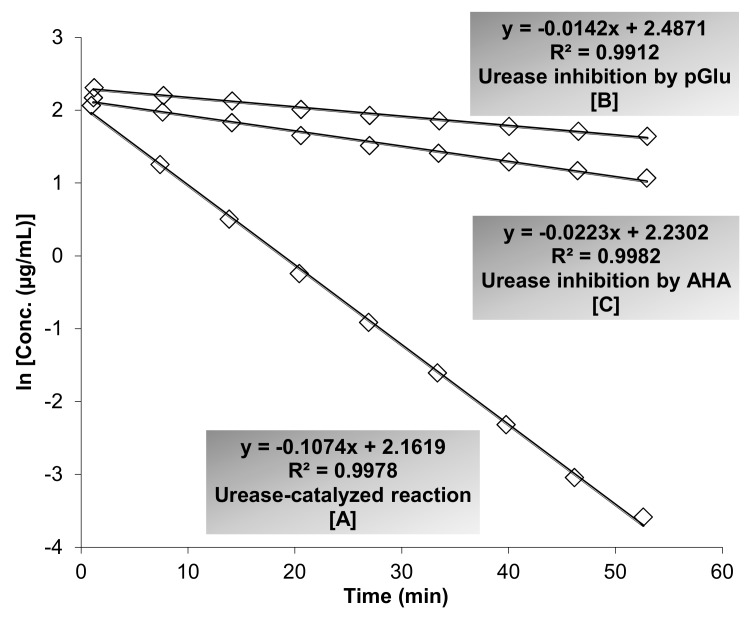
The Electrospray Ionization-Mass Spectrometry (ESI-MS) based method was employed to determine the inhibitory action of pyroglutamic acid (pGlu) and acetohydroxamic acid (AHA) against urease. As shown, the slopes represent the reaction rate constant (RRC), where k_0_ is the RRC of urease-catalyzed reaction (k_0_ = 0.1074/min) [A] and k is the RRC of urease-catalyzed reaction inhibited by pGlu (k = 0.0142/min) [B] and AHA (k = 0.0223/min) [C]. Fluctuations of urea concentrations are displayed as logarithms of concentration. The relative standard deviation (RSD; %) of multiple measured slopes (less than 10%) was calculated to precisely the time–course analysis. For clarity of the presented figure, multiple measurements have not been manifested. The half-maximal inhibitory concentration (IC_50_) values for pGlu and AHA were recorded to be 1.8 and 3.9 μM, respectively.

The inhibitory action of pGlu and AHA against JBU was studied by employing the ESI-MS-based method. As presented in Figure 1, the reaction rate constant (RRC) in the presence of inhibition (k) is lower than the RRC of urease-catalyzed reaction (in the absence of inhibition; k_0_), where k in the presence of pGlu (k = 0.0142/min) and AHA (k = 0.0223/min) and (k_0_ = 0.1074/min). IC_50_ values were assessed following the procedure described by Hassan et al. [26]. The IC_50_ values for pGlu and AHA were recorded to be 1.8 and 3.9 μM, respectively.

It is known that the inhibition of urease has beneficial implications in agriculture, animal, and human health via reducing the amount of ammonia released by the inhibition of the urease-catalyzed reaction. Consequently, numerous natural, synthetics, and semi-synthetic compounds were investigated for their inhibitory properties against urease [45,46]. Urease inhibitors (UIs) are acknowledged to be powerful antiulcer drugs that regulate the negative outcome of ureolytic bacterial infections in humans and animals. Over the past few decades, many UIs were discovered with various degrees of inhibition properties. Unfortunately, most of these inhibitors were prohibited from being utilized in vivo due to their toxicity or instability. Therefore, the search for new sources of natural UIs that afford low toxicity, lessened side effects, bioavailability, and better stability has acquired high precedence to overwhelm such complications [46,47]. Several imperative review articles have critically reviewed and documented various natural, synthetic, and semi-synthetic UIs studied on various types of ureases and performed in vitro and in vivo studies. These articles have covered a wide range of chemical classes including phenolic compounds, alkaloids, terpenoids, and other synthetic molecules [46,47,48,49]. Given the potential efficacy of such substances against urease, it is valuable to highlight that the problem associated with the toxicity and instability, which were observed in many in vivo investigations, is a challenge for many researchers and pharmaceutical industries worldwide. To the best of our knowledge, this is the first study on the inhibitory effect of pGlu on urease.

### 3.4. Evaluation of Cytotoxicity Characteristics 

In this study, we evaluated the cytotoxic effect of pGlu on human cervical carcinoma cells (HeLa-R2) and normal human fetal lung fibroblast cells (MRC-5) in comparison with the standard anticancer drug cisplatin. The obtained results advocated that the treatment of HeLa-R2 cells with pGlu displayed very low cytotoxicity (IC_50_ = 96.42 µg/mL) compared with cells treated with cisplatin (IC_50_ = 6.32 µg/mL) (Table 3). Additionally, very low cytotoxicity was observed when MRC-5 cells were treated with pGlu (IC_50_ = 97.21 µg/mL) compared with cells treated by cisplatin (IC_50_ = 9.14 µg/mL). Based on the obtained results, pGlu displayed a good level of safety profile, where no significant cytotoxic effect on both HeLa-R2 and MRC-5 cells were noted. This finding could be the first step towards further investigations that might validate the practical use of pGlu as a non-prescription dietary supplement.

The main characteristic of an effective drug is to reach its desired target in the body in an active form with adequate concentration and no toxicity. Consequently, the evaluation of potential cytotoxicity of any test drug is an essential procedure [50]. Despite the low toxicity induced by pGlu at specific concentrations, it has been previously described that pGlu in high levels can act as an acidogen with the ability to cause acidosis and a metabotoxin that can result in adverse health effects [51]. Persistently high levels of pGlu detected in human blood are allied with several inborn errors of metabolism including propionic acidemia, 5-oxoprolinase deficiency, 5-oxoprolinuria, glutathione synthetase deficiency, hawkinsinuria, hemolytic anaemia, and central nervous system dysfunction [51,52,53]. In addition, high anion gap metabolic acidosis was observed when high levels of pGlu distinguished in blood following acetaminophen overdose [5]. Therefore, additional in vivo and clinical studies should be performed to determine the optimal concentration or dose of pGlu that could reach the desired target with the least possible cytotoxic effect.

### 3.5. Evaluation of Molecular Docking Analyses 

In recent years, computational chemistry has played a dynamic role in drug design and discovery research as an important tool for predicting the possible interaction of any test ligand with putative receptors, which, in turn, could aid researchers to select better drug candidates for further in vitro investigations [54]. In our study, we evaluated the binding mode and molecular interaction of pGlu with the active sites of PDE5A1, ACE, and urease (JBU) along with predicted structure–activity relationships using PyRx docking software associated with Autodock VINA along with Discovery studio visualizer program.

#### 3.5.1. Interaction of pGlu with Actice Site of PDE5A1

**Figure 2:**
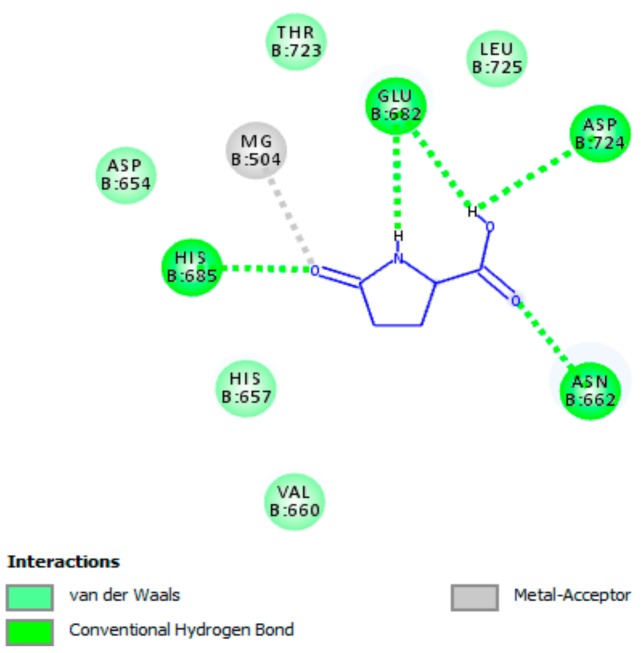
A two-dimensional diagram illustrates the interaction of pyroglutamic acid (pGlu) with the active site of phosphodiesterase 5A1 (PDE5A1). Essential interactions with residues of the active site that are crucial for inactivating the enzyme are presented. As shown, pGlu interacts with magnesium ion in the M site (metal-binding site) and interacts with other important residues in the L region (lid region) of the active site of the enzyme.

The docking results showed that docking scores for pGlu and sildenafil, which are displayed as binding affinities, were observed to be −7.1 and −7.9 kcal/mol, respectively. As elicited in Figure 2, pGlu remarkably bound to the active site of PDE5A1, which in turn resulted in obvious PDE5A1 inactivation. A number of essential interactions that play a central role in enzyme inhibition were observed, including hydrogen bonding interactions, van der Waals interactions, as well as an interaction with magnesium ion in the metal-binding site. The detected amino acid residues were observed earlier to be significant residues in the active site of the enzyme and are responsible for enzyme suppression [23]. Considering the remarked binding affinity of pGlu with the metal-binding site and lid region of the active site of the enzyme, we may suppose pGlu as a competitive inhibitor of PDE-5. 

#### 3.5.2. Interaction of pGlu with Actice Site of ACE

**Figure 3:**
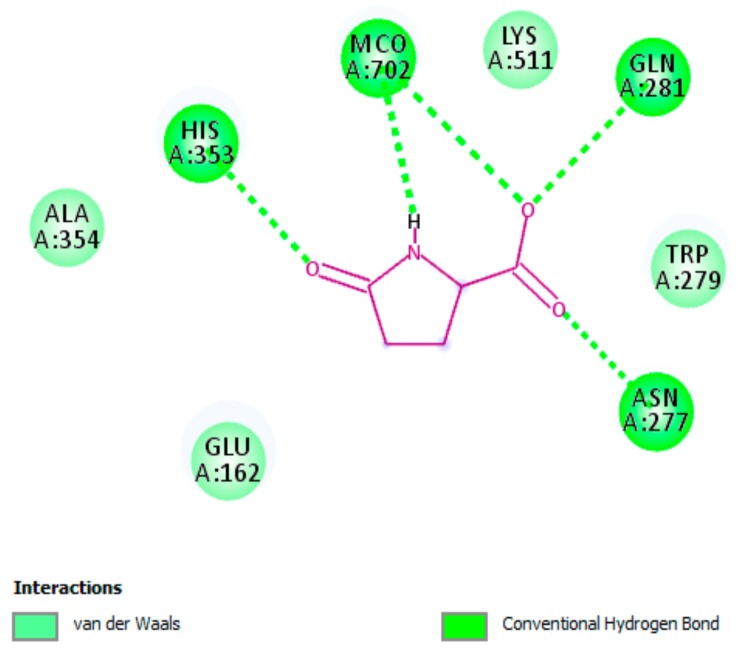
A two-dimensional interaction scheme of pyroglutamic acid (pGlu) with the catalytic site of angiotensin-converting enzyme. Essential hydrogen bonding interactions along with van der Waals interactions with residues of the catalytic site, which are crucial for inactivating the enzyme, are shown.

Docking scores (expressed as binding affinities) for pGlu and captopril were perceived to be −5.3 and −6.1 kcal/mol, respectively. The obtained results indicated that pGlu interacts with the active site of ACE by establishing hydrogen bonding interactions and van der Waals interactions with important residues of ACE active sites (Figure 3). These interactions are vigorous for suppressing ACE activity. As exposed in Figure 3, all amino acid residues were earlier defined in the active site of human ACE and were noticed to be pivotal for the stabilization of the enzyme [55]. It has been confirmed that ACE is a zinc-containing enzyme, which means that zinc ion is indispensable for the catalytic activity of the enzyme. Interestingly, pGlu was found to bind to the different active pocket of the enzyme and no interactions were observed with zinc active sites. This indicates that pGlu is not competing with the substrate, and consequently acts as a non-competitive inhibitor by avoiding the formation of enzyme-product complexes.

#### 3.5.3. Interaction of pGlu with Actice Site of Urease

**Figure 4:**
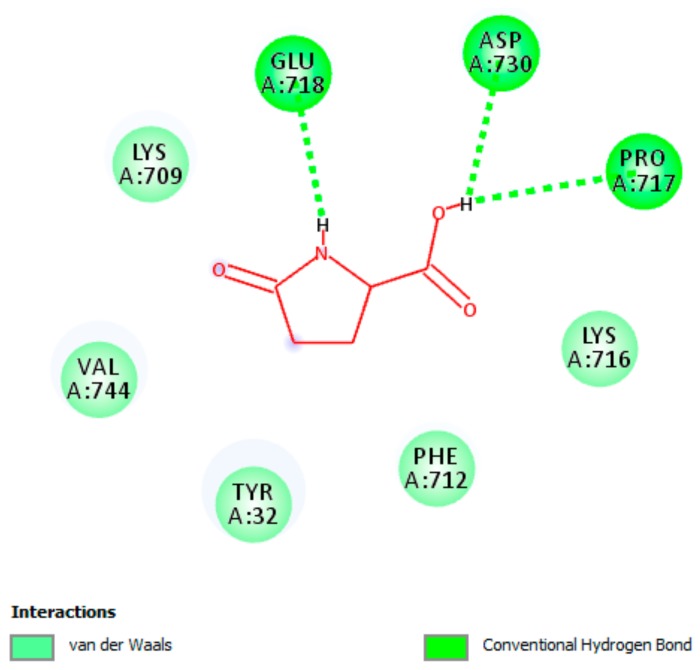
A two-dimensional illustration shows pyroglutamic acid (pGlu) binds to the catalytic pocket of Jack bean urease. Essential hydrogen bonding interactions along with van der Waals interactions with residues of the catalytic pocket, which are crucial for inactivating the enzyme, are shown.

Docking score (expressed as binding affinity) for pGlu was detected to be −4.8 kcal/mol. The docking analysis disclosed that the best orientation of pGlu in the active pocket of JBU was designed by creating hydrogen bonding interactions as well as van der Waals interactions with essential residues of the active pocket of JBU (Figure 4). All interacted amino acid residues were hitherto reported to be critical for JBU stabilization [28,56,57]. The docking results did not confirm any interactions of pGlu with the active-site Ni^2+^, which indicates that pGlu bound to a different catalytic pocket of JBU. Accordingly, we may suggest that pGlu as non-competitive inhibitor of JBU, and hence does not compete with the substrate. 

## 4. Conclusions

There is an ongoing necessity for an exploration or development of new enzyme inhibitors that combine high efficacy, low toxicity, reduced undesirable effects, and various mechanisms of action. 

The present study investigated the unexplored pharmacological effects of pGlu using various analytical methods, where this natural molecule has shown to elicit remarked inhibitory property against the key enzyme PDE5A1, involved in the treatment of erectile dysfunction and pulmonary arterial hypertension (refer to Results and Discussion section). Additionally, pGlu was detected to efficiently inactivate ACE and urease (refer to Results and Discussion section), where these two enzymes play a central role in many clinically significant syndromes. Besides, pGlu showed a putative level of safety profile based on the obtained cytotoxicity results, which might pave the road for additional studies to approve its practical use as a dietary supplement. Eventually, additional investigations need to be performed using modified delivery systems to reduce the potential adverse actions of pGlu prior to its probable practical application. Some other important studies are required to authenticate the reported activities in vivo and in clinical trials, along with the evaluation of pharmacokinetic and pharmacodynamic characterizations.

## Figures and Tables

**Table 1:** Anti-enzymatic activities of pyroglutamic acid and sildenafil citrate against the human catalytic domain of phosphodiesterase-5A1.

Inhibitor	IC_50_ (µM)
pGlu	5.23 ± 1.33
Sildenafil citrate	7.14 ± 1.52

PRISM software version 8.0 (GraphPad Software, Inc., La Jolla, CA, USA) was used for statistical analysis. Values displayed are means ± standard deviation (SD) of three independent experiments achieved in triplicate. PDE5A1: Phosphodiesterase-5A1; pGlu: Pyroglutamic acid; IC_50_: Half-maximal inhibitory concentration.

**Table 2:** Inhibitory effects of pyroglutamic acid and captopril on the human angiotensin-converting enzyme.

Inhibitors	% Inhibition
pGlu	98.2 ± 1.12
Captopril	99.6 ± 1.64
ACE-catalyzed reaction (no inhibition)	Nd

PRISM software version 8.0 (GraphPad Software, Inc., La Jolla, CA, USA) was used for statistical analysis. Values are displayed as the mean ± standard deviation (SD) (*n* = 3). ACE: Angiotensin-converting enzyme; pGlu: Pyroglutamic acid; Nd: Not determined.

**Table 3:** Cytotoxic effect of pyroglutamic acid and cisplatin on human cervical carcinoma cells and normal human cells.

	IC_50_ (µg/mL)	
Compound	HeLa-R2	MRC-5
pGlu	96.42 ± 0.92	97.21 ± 0.61
Cisplatin	6.32 ± 0.62	9.14 ± 0.42

PRISM software version 8.0 (GraphPad Software, Inc., La Jolla, CA, USA) was used for statistical analysis. Values presented are means ± standard deviation (SD) of three individual measurements assayed in triplicate. HeLa-R2: Human cervical carcinoma cells; MRC-5: Normal human fetal lung fibroblast cells; pGlu: Pyroglutamic acid; IC_50_: The concentration of molecule that exhibits 50% suppression of cell survival. ANOVA followed by post-hoc comparison tests (Dunnett and Student-Newman-Kuels) were used to determine the differences between treatments with test compounds and positive control. Statistical significance was *p* < 0.05.
